# A double-blind, randomized, placebo-controlled, single-center clinical trial of jiaotaiwan for the treatment of insomnia symptoms caused by disharmony of the heart and kidney

**DOI:** 10.3389/fphar.2022.1011003

**Published:** 2022-11-04

**Authors:** Nengzhi Xia, Chengrou Jiang, Yiwei Zhou, Qun Huang, Lufeng Hu, Haihuan Zeng, Lin Luo, Zhengzhong Yuan

**Affiliations:** ^1^ Department of Radiology, The First Affiliated Hospital of Wenzhou Medical University, Wenzhou, China; ^2^ Wenzhou Medical University, Wenzhou, China; ^3^ Department of Traditional Chinese Medicine, The First Affiliated Hospital of Wenzhou Medical University, Wenzhou, China; ^4^ Department of Pharm, The First Affiliated Hospital of Wenzhou Medical University, Wenzhou, China; ^5^ Sleep Monitoring Center, The First Affiliated Hospital of Wenzhou Medical University, Wenzhou, China; ^6^ Institute of Basic Research in Clinical Medicine, China Academy of Chinese Medicine Sciences, Beijing, China

**Keywords:** insomnia, jiaotaiwan, traditional herbal medicine, disharmony of the heart and kidney, randomized controlled trial

## Abstract

**Background:** Jiaotaiwan (JTW) is a classical tranquillizing prescription in traditional Chinese medicine (TCM) for the treatment of insomnia symptoms caused by disharmony of the heart and kidney (ISDHK). This study aimed to evaluate the effectiveness and safety of JTW for treating ISDHK in a double-blind, randomized, placebo-controlled trial.

**Methods:** From September 2018 to February 2020, 128 participants with ISDHK were included in this single-center clinical trial. All participants were equally and randomly divided into either the JTW group (2-g JTW granules, b.i.d. for 7 days) or placebo group (2-g placebo granules, b.i.d. for 7 days). Pittsburgh Sleep Quality Index (PSQI) scores were set as the primary outcome, and polysomnography (PSG), ^1^H-magnetic resonance spectroscopy (^1^H-MRS), blood tests, and Disharmony of Heart and Kidney Scoring System (DHKSS) and clinical global impression (CGI) scores were used as secondary outcomes. Laboratory tests were used to evaluate the safety of JTW. All data were collected at baseline and posttreatment.

**Results:** A total of 106 participants completed this clinical trial. Symptom relief was more apparent in the JTW group than the placebo group (PSQI total score: 9.34 ± 3.578 vs. 10.98 ± 3.073, respectively; *p* = 0.006). However, no PSG changes were observed between the two groups (*p* > 0.05). Higher CGI and lower DHKSS scores were observed after JTW treatment. Serum melatonin was increased in patients with ISDHK after JTW treatment (JTW, 339.09 ± 256.894 vs. placebo, 219.59 ± 169.045; *p* = 0.004). There were significant posttreatment differences in metabolites in the left cerebellum between the two groups (myoinositol: JTW, 13.47 ± 2.094 vs. placebo, 12.48 ± 2.449; *p* = 0.021; choline: JTW, 3.96 ± 0.657 vs. placebo, 3.65 ± 0.562; *p* = 0.008). In terms of safety, JTW had no noticeable adverse effects relative to placebo.

**Conclusion:** JTW was effective and well tolerated for the treatment of ISDHK. The development of large-scale trials with longer follow-up durations is recommended to provide further evidence.

**Clinical Trial Registration:** clinicaltrials.gov, identifier ChiCTR1800019239

## 1 Introduction

Insomnia is the most common sleep problem in the general population and seriously affects not only people’s physical and mental health but also their quality of life and work efficiency (Patel et al., 2018; Sutton, 2021). Clinical epidemiological studies have suggested that insomnia is associated with hypertension, stroke, depressive disorders, psychotic disorders, dementia, substance abuse disorders and weakened immunity (Yin et al., 2017; Sanjari Moghaddam et al., 2021). The aetiology and pathophysiology of insomnia are complex and involve genetic, environmental, behavioural, and physiological factors (Buysse, 2013). Therefore, the treatments of insomnia can be complex and time consuming and mainly include behavioural, cognitive, and Western pharmacological interventions (such as benzodiazepine receptor agonists) (Buysse, 2013; Kay-Stacey and Attarian, 2016). However, none of these therapies is effective in the long term because of the difficulty of effective implementation or limitations of efficacy and adverse reactions. Recently, traditional Chinese medicine (TCM) has been found to be an effective complement and alternative for treating insomnia (Yeung et al., 2012; Ng and Parakh, 2021).

Chinese herbal medicine (CHM), as either single herbs or herbal formulas, has been widely used for the treatment of insomnia in China. The importance of CHM is evidenced by approval in the latest evidence-based guidelines in China for insomnia (Liu, 2011.). In recent years, the use of CHM has increased in the Western world, although the mechanism by which CHM improves sleep remains undefined (Frass et al., 2012; Leach and Page, 2015). However, a systematic review of CHM for insomnia showed that only eight studies had a Jadad score ≥ 3 among 217 randomized controlled trials (RCTs), and seven of these studies had a high risk of bias in at least one domain (Yeung et al., 2012). The authors considered that the current evidence was insufficient to support the efficacy of CHM for insomnia due to the poor methodological quality of the studies. Another systematic review also revealed that the efficacy of CHM for insomnia remained unclear due to heterogeneity and that the individual CHM formula for insomnia deserves further study (Ni et al., 2015). Therefore, it is necessary and urgent to conduct clinical trials and obtain high-level evidence to confirm the curative effect of CHM for insomnia.

TCM theory considers sleep to be a process of harmony between Yin and Yang works as Yang enters Yin leads to sleep and Yang comes out of Yin leads to wakefulness. Insomnia is considered a result of disharmony between Yin and Yang. Based on database searching and multi-center study results, insomnia symptoms caused by disharmony of the heart and kidney (ISDHK) are the dominant type of insomnia (Yuan et al., 2011; Poon et al., 2012). Disharmony of the heart and kidney mainly refers to the pathological phenomenon of abnormal physiological coordination between heart yang and kidney yin. Under normal condition, heart yang and kidney yin coordinate with each other, restrict each other, traffic each other, maintain dynamic balance. When the kidney Yin deficit, Yin essence cannot flow up to co-work with heart Yang then heart Yang will be too hyperactivity to descend into kidney yin. Such condition is called disharmony of the heart and kidney, which is a mainly cause of insomnia. From the perspective of yin and yang, the disharmony of the heart and kidney can also be called yin deficiency and excessive fire (Chen et al., 2016). According to TCM theory, Jiaotaiwan (JTW), a classical tranquillizing formula, has the effect of harmonizing the communication between the heart and kidney and is considered one of the most commonly used CHMs for insomnia (Yeung et al., 2012). JTW consists of Rhizome Coptidis and Cortex Cinnamomi (Committee of the Pharmacopoeia of China, 2020). Modern pharmacological studies have shown that JTW has sedative-hypnotic and antidepressant effects, as well as hypoglycaemic, lipid-lowering and anti-inflammatory effects (Zou et al., 2017; Su et al., 2020). JTW has been considered helpful for insomnia symptom relief, but there is still a lack of adequately powered studies to provide evidence-based medicine. Therefore, the present double-blind, randomized, placebo-controlled single-center clinical trial was designed to accurately determine the effectiveness and safety of JTW for treating ISDHK and provide a reliable basis for clinical application.

## 2 Materials and methods

### 2.1 Study design

This study was a double-blind, randomized and placebo-controlled trial with a 1:1 allocation ratio that evaluated the efficacy and safety of JTW relative to placebo in treating ISDHK and was conducted at the First Affiliated Hospital of Wenzhou Medical University. This study was approved by the Hospital Institutional Review Board, and signed informed consent was obtained from each participant. In our recent study, the study protocol was described in detail (including randomization, blinding, sample size calculation, and withdrawal criteria) (Zeng et al., 2020), which strictly followed the Consolidated Standards of Reporting Trials (CONSORT) guidelines. This trial was registered with the Chinese Clinical Trial Registry (ChiCTR1800019239).

### 2.2 Participants

A total of 128 participants with ISDHK were included from the Sleep Center and Traditional Chinese Medicine Department of our Hospital between September 2018 and February 2020. There were 50 males and 78 females, and their mean age was 39.21 ± 10.76 years. All participants were equally and randomly divided into the JTW group (*n* = 64) or placebo group (*n* = 64). The participants were asked to complete the Pittsburgh Sleep Quality Index (PSQI), to undergo polysomnography (PSG), ^1^H-magnetic resonance spectroscopy (^1^H-MRS), and laboratory tests, and to be evaluated by the Disharmony of Heart and Kidney Scoring System (DHKSS) and clinical global impression (CGI) at baseline and posttreatment. Information regarding other clinical characteristics, including sex, age, race, marital status, job, educational level, income status, smoking and alcohol consumption status, and other previous history, were collected.

The inclusion criteria for participants with ISDHK were as follows: (1) aged 18–60 years, with an education level of junior high school or above; (2) met the diagnostic criteria for insomnia in the Chinese Classification and Diagnostic Criteria of Mental Disorders, Version 3 (CCMD-3) ( Psychosis Branch of Chinese Medical Association, 2001); (3) had a total PSQI score ≥ 7; (4) met the diagnostic criteria for ISDHK according to the Internal Medicine of TCM; and (5) had a total DHKSS score ≥ 9. The exclusion criteria for participants with ISDHK were as follows: (1) secondary insomnia or insomnia caused by lifestyle or environmental changes; (2) psychiatric disorder or other somatic disorders affecting the central nervous system; (3) abnormal liver and kidney function caused by heart, kidney, liver and haematopoietic system diseases; (4) use of drugs for sleep disorders (the past week) or antipsychotic medications (the last month); and (5) alcohol abuse, allergies, pregnancy or lactation.

### 2.3 Randomization and allocation concealment

Randomization was done by a trial administrator who was not involved in the clinical intervention or evaluation. A sequence of labels (A or B) at 1:1 ratio was generated with block randomization, numbered in order. The trial intervention packages with the same plastic packages were prepared by the pharmaceutical company according to the labeled sequence. The study coordinator provided the numbered package of intervention according to the visiting time sequence of the participants at baseline. The generated sequence of the group assignment and numbers will be sealed in opaque envelopes and stored in double-locked cabinets. All participants, doctors, statisticians, trial administrator and coordinator in this research were blinded to the allocation sequence throughout the entire trial phase.

### 2.4 Intervention

JTW has the effect of harmonizing the communication between the heart and kidney and commonly used for ISDHK in Eastern countries for centuries. The participants were asked to ingest placebo or JTW granules (2 g, b.i.d. at 4:00 p.m. and 9:00 p.m.) for 1 week. Based on results in our previous animal experiments and clinical experience, the duration of 1 week was chosen to evaluate the effectiveness of JTW (Ni et al., 2019; Zeng et al., 2020). A total of 25 major chemical components of JTW were identified in our previous study (Lin et al., 2022).

The JTW consisted of JTW soft extract (1.1 g) and the excipient of corn starch (0.88 g). The soft extract was made of Rhizome Coptidis (the dried root of Coptis chinensis Franch [Ranunculaceae], containing over 5% berberine and 3.3% total amount of epiberberine, coptisine and palmatine by weight, 10 g) and Cortex Cinnamomi (the dried stem bark of Cinnamomum aromaticum Nees [Lauraceae], containing Cinnamic aldehyde not less than 1.5% by weight and volatile oil not less than 1.2% (ml/g), 1 g) dissolved in water. JTW was prepared as follows: Rhizome Coptidis slices were decocted with water and filtered. The filtrate was concentrated into clear paste (the extraction rate of dry extract was 12–22%) and dried. The excipient was added and mixed, then processed into granules. 1 g formula granules was equivalent to 4.5 g prepared slices. Cortex Cinnamomi slices were decocted with water. The volatile oil was extracted and filtered. The filtrate was concentrated into clear paste (the extraction rate of dry extract was 4–8%). The volatile oil inclusion compound was added and dried. The excipient was added and mixed, then processed into granules. 1 g formula granules was equivalent to 5.5 g prepared slices. Rhizome Coptidis and Cortex Cinnamomi were mixed in the proportions of 10:1 and processed in a production line in JTW workshop. Rhizome Coptidis produced in Sichuan Province, Cortex Cinnamomi in Guangdong Province. The placebo contained corn starch, citric acid, caramel colour and lactose hydrate. The empty wrapping papers were retrieved and used to ensure medication compliance.

The Beijing Kangrentang Pharmaceutical Co., Ltd. (Beijing, China) provided both placebo and JTW with the same weight, appearance and colour, which were quality controlled by their quality management department in accordance with the granule section of the Pharmacopoeia of the People’s Republic of China (2015 edition). The voucher species were stored at Beijing key Laboratory of production process Control and quality Evaluation of traditional Chinese Medicine of Beijing University of Chinese Medicine.

### 2.5 Outcome assessment

#### 2.5.1 Primary outcome measure

Pittsburgh Sleep Quality Index (Buysse et al., 1989): The PSQI was used to subjectively evaluate the sleep quality of subjects in the previous month. The PSQI is a questionnaire consisting of 19 self-rated items with a total score that can range from 0 to 21. The higher the score was, the worse the sleep quality. The PSQI consists of seven subscales: sleep quality, sleep latency, sleep duration, habitual sleep efficiency, sleep disturbances, use of hypnotics, and daytime dysfunction. The PSQI total score was designed as the primary outcome.

#### 2.5.2 Secondary outcome measures

Polysomnography: PSG was performed with the Alice 5 PSG System (Philips Respironics, Pennsylvania, United States), which included six channels (F3, F4, C3, C4, O1 and O2) of electroencephalogram, electrooculography (placed at bilateral lateral canthus), and submental electromyography. PSG was carried out in the sleep monitoring laboratory of the Sleep Center, which was quiet, undisturbed, comfortable and maintained at a constant temperature of 20°C–25°C. All the recorded data were automatically analysed and processed using the associated Sleepware software and then manually corrected by the sleep technician. The PSG parameters mainly included total sleep time, sleep efficiency, sleep latency, rapid eye movement (REM) stage latency, and the durations spent in particular sleep stages (such as N1, N2, and N3).

^1^H-MRS acquisition and postprocessing: A 3.0 T magnetic resonance imaging (MRI) system (Achieva, Philips, Best, Netherlands) was used to perform MR examinations with an 8-channel head coil. The patients lay supine on the examination bed with their head inside the scanner. The ^1^H-MRS data were collected using a point-resolved spectroscopy (PRESS) sequence (echo time (TE) = shortest; repetition time (TR) = 2000 m s; spectral bandwidth: 2000 Hz; data points: 1024; measurement time: 4 min 52 s). Regions of interest (ROIs) were placed in the bilateral prefrontal lobes, hippocampi, cerebellum and anterior cingulate gyri. The acquired spectra were fitted and quantified with LCModel using the corresponding basis spectra (GAMMA_PRESS_TE38_128MHZ_806D.BASIS) according to the scan parameters. The concentrations of major detectable brain metabolites were obtained, including N-acetylaspartate (NAA), choline (Cho), creatine (Cr), glutamine and glutamate (Glx), myoinositol (mI) and glutathione (GSH).

Blood tests: The levels of N-acetyl-5-methoxytryptamine (melatonin), adenosine, gamma-aminobutyric acid (GABA) and adrenocorticotropic hormone (ACTH) were detected in blood samples. Venous blood was collected at 7:00 a.m. (for adenosine, GABA and ACTH) and 9:30 p.m. (for melatonin). Melatonin levels were assessed with human melatonin enzyme-linked immunoassay kit (CUSABIO, Wuhan, China). ACTH levels were measured by chemiluminescent immunoassay (Cobas 8000, Roche Diagnostics, Mannheim, Germany). Adenosine and GABA levels were assessed with ultra-performance liquid chromatography-tandem mass spectrometry (UPLC-MS/MS; Waters Corp, Milford, MA, United States). Adenosine or GABA samples were precipitated by 300 μl acetonitrile (0.1% formic acid) and centrifuged at 12000r•min-1 for 5min, after that, the supernatant was transferred for UPLC-MS/MS analysis, with an injection volume of 2 μl. The GABA levels were detected at m/z 104.00 →87.10 and the adenosine levels were detected at m/z 268.15 →136.00 in the multiple reaction monitoring (MRM) mode with an ESI ion source in positive ionization mode. UPLC-MS/MS data were acquired by Masslynx 4.1 software (Waters Corp.).

Disharmony of heart and kidney scoring system: The DHKSS includes the main symptoms, accompanying symptoms, tongue features and pulse features. The main symptoms consist of difficulty falling asleep, dreaminess, sleep fragmentation, early awakening, awakening and being unable to fall asleep again at night. The accompanying symptoms include upset, palpitation, dizziness, tinnitus, sour waist, soft legs, hands and feet heart heat, hot flashes, night sweats, dry mouth and pharynx, and seminal emission/irregular menstruation. The higher the total score was, the worse the ISDHK. The scoring of the main symptoms used a 4-point scale (0, 3, 6, and 9) and was rated depending on the severity of the insomnia (0 = none, 9 = very severe); the scoring of accompanying items used a scale of 0–4 points based on their frequency.

Clinical global impression: The CGI represents the physician’s overall clinical impression of ISDHK subjects. Improvements in or worsening of insomnia was evaluated using an 8-point scale (0–7) according to the changes from before to after treatment. The CGI is rated on the following scale: 0, not evaluated; 1, significant improvement; 2, moderate improvement; 3, slight improvement; 4, no change; 5, slight deterioration; 6 moderate deterioration; and 7, severe deterioration.

### 2.6 Safety and adverse events monitoring

At the end of the clinical trial, a routine physical examination was performed for each participant, which included the collection of weight, temperature, heart rate, breath rate and blood pressure. All adverse events were documented, and all suspicious events were closely monitored throughout the study. The participants were encouraged to report any symptoms or signs. Additionally, laboratory tests (routine urine, routine blood test and blood biochemical tests) and electrocardiograms were taken at baseline and posttreatment.

### 2.7 Statistical analysis

Intention-to-treat analysis (ITT) was used for data analysis. Last observation carried forward (LOCF) method was used to estimate missing values of major variables. Numerical data are described as the mean ± standard deviation (SD), and independent samples t tests or Wilcoxon rank sum tests were used for the group comparison according to normality. Counts and percentages were used to present categorical data, which were compared with chi-square tests. *p* < 0.05 was considered to indicate a statistically significant difference. All the statistical analysis was performed with SAS software (Version 9.4, SAS Institute, Cary, North Carolina, United States).

## 3 Results

**FIGURE 1:**
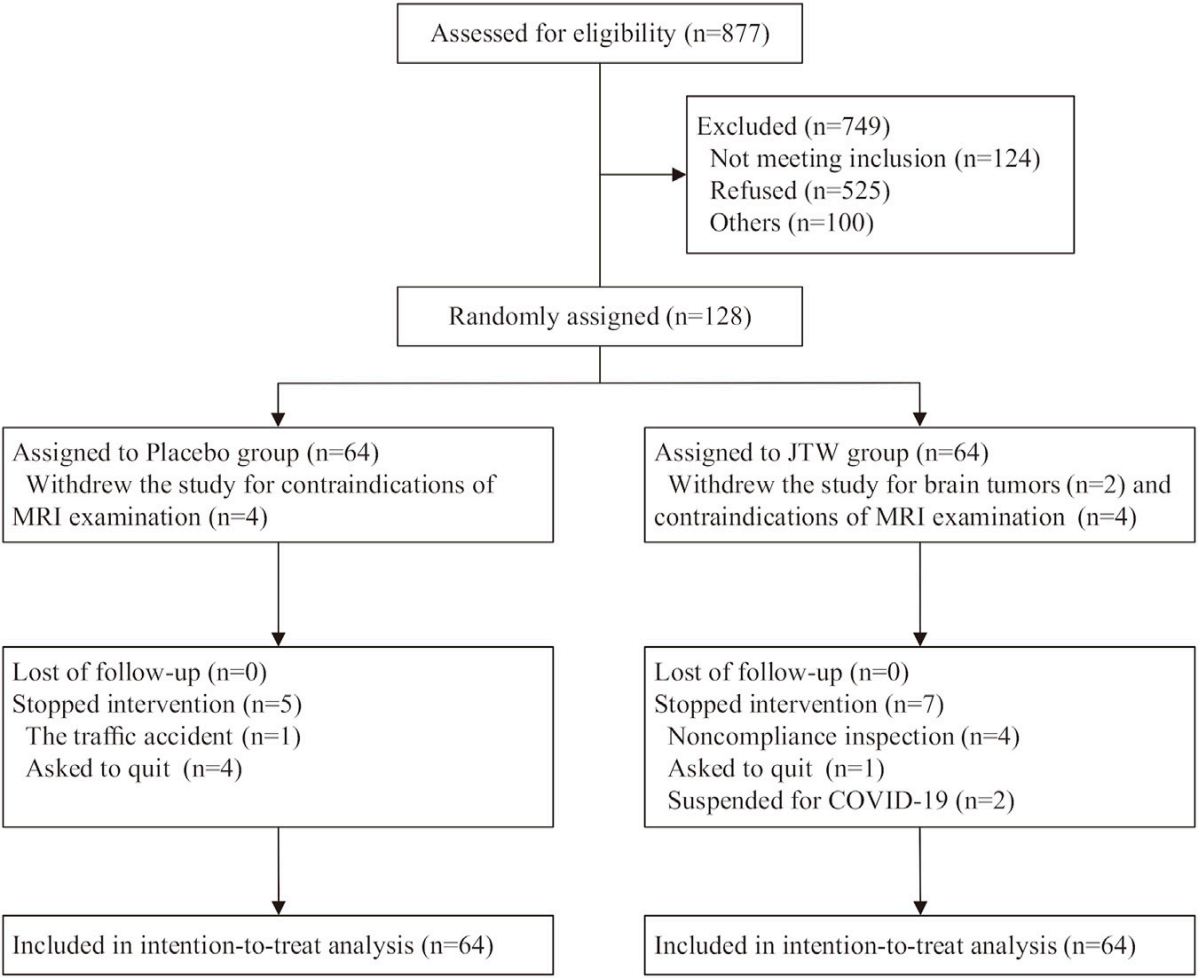
The enrollment flow diagram.

Patient flow through the trial. A detailed illustration of the enrolment flow diagram through the trial is shown in Figure 1. A total of 128 participants were randomized to the JTW group (*n* = 64) or to the placebo group (*n* = 64). There were 106 subjects completed the entire trial with 55 participants in the placebo group and 51 participants in the JTW group. It was worth mentioning that the data of MRS and PSG were missing for the 10 participants who abandoned the trial due to metal artifacts or brain tumors on MRI (4 subjects in the placebo group and 6 subjects in the JTW group). The number of participants included for data analysis were 60 participants in the placebo group and 58 participants in the JTW group at baseline and posttreatment for MRS and PSG (Tables 3, 4).

### 3.1 Demographic and clinical characteristics of the participants

As shown in Table 1, there were no significant differences in demographic characteristics, including age, sex, nationality, marital status, occupation, education, and income status between the two groups at baseline. There were more females than males (62.50% in the JTW group and 59.38% in the placebo group), while the difference showed no statistically significant between the two groups. In addition, no significant differences were observed in clinical features, including smoking status, and drinking status.

**TABLE 1:** Demographic and clinical characteristics of the participants included in the JTW and placebo groups.

	Placebo	JTW	*p* Value
Number	64	64	—
Age (years)	38.56 ± 10.959	38.89 ± 10.538	0.863
Sex			0.7171
Male	26 (40.63%)	24 (37.50%)	
Female	38 (59.38%)	40 (62.50%)	
Nationality			0.5591
Han Nationality	62 (96.88%)	63 (98.44%)	
Others	2 (3.13%)	1 (1.56%)	
Marital status			0.1594
Single (include divorced)	14 (21.88%)	17 (26.56%)	
Married	50 (78.13%)	44 (68.75%)	
Others	0 (0.00%)	3 (4.69%)	
Occupation			0.6973
Worker	8 (12.50%)	8 (12.50%)	
Farmer	1 (1.56%)	5 (7.81%)	
Administrative worker	7 (10.94%)	7 (10.94%)	
Service industry	20 (31.25%)	15 (23.44%)	
Intellectuals	4 (6.25%)	6 (9.38%)	
Freelancer	16 (25.00%)	16 (25.00%)	
Others	8 (12.50%)	7 (10.94%)	
Educational level			0.7257
Junior middle school	20 (31.25%)	17 (26.56%)	
Senior high school	10 (15.63%)	15 (23.44%)	
University	29 (45.31%)	27 (42.19%)	
Graduate school	5 (7.81%)	5 (7.81%)	
Smoking status			0.7525
Yes	6 (9.38%)	5 (7.81%)	
No	58 (90.63%)	59 (92.19%)	
Alcohol consumption status			0.8179
Yes	11 (17.19%)	12 (18.75%)	
No	53 (82.81%)	52 (81.25%)	
Income status (RMB/month)			0.6812
< 1000	11 (17.19%)	8 (12.50%)	
1000–3000	9 (14.06%)	6 (9.38%)	
3000–6000	23 (35.94%)	25 (39.06%)	
> 6000	21 (32.81%)	25 (39.06%)	

### 3.2 Primary outcome evaluation of jiaotaiwan for insomnia symptoms caused by disharmony of the heart and kidney

After JTW treatment, the total PSQI score in patients with ISDHK was significantly decreased (*p* = 0.006), as shown in Table 2. This demonstrated effective improvements in some subscales of the PSQI, including sleep quality, sleep latency, sleep time and sleep efficiency.

**TABLE 2:** Comparison of PSQI scores in the participants at baseline and posttreatment between the JTW and placebo groups.

	Placebo	JTW	*p* Value
Number			
Baseline	64	64	—
Posttreatment	64	64	—
Sleep quality			
Baseline	2.44 ± 0.588	2.39 ± 0.581	0.651
Posttreatment	2.00 ± 0.735	1.70 ± 0.749	0.025
Sleep latency			
Baseline	2.36 ± 0.804	2.50 ± 0.873	0.345
Posttreatment	2.30 ± 0.749	1.95 ± 0.862	0.018
Sleep time			
Baseline	1.83 ± 1.001	1.92 ± 1.013	0.599
Posttreatment	1.75 ± 0.976	1.38 ± 0.968	0.031
Sleep efficiency			
Baseline	2.13 ± 1.134	2.34 ± 1.011	0.252
Posttreatment	2.08 ± 1.159	1.66 ± 1.185	0.044
Sleep disturbances			
Baseline	1.61 ± 0.553	1.63 ± 0.604	0.879
Posttreatment	1.47 ± 0.534	1.36 ± 0.574	0.266
Sleep medications			
Baseline	0.44 ± 0.974	0.34 ± 0.821	0.557
Posttreatment	0.56 ± 1.022	0.30 ± 0.749	0.098
Daytime dysfunction			
Baseline	1.23 ± 0.972	1.38 ± 0.900	0.397
Posttreatment	0.83 ± 0.918	1.00 ± 0.836	0.270
Total score			
Baseline	12.03 ± 2.823	12.50 ± 2.971	0.362
Posttreatment	10.98 ± 3.073	9.34 ± 3.578	0.006

### 3.3 Secondary outcome evaluation of jiaotaiwan for insomnia symptoms caused by disharmony of the heart and kidney

Table 3 shows that no differences were observed in the PSG measures between the treatment and placebo groups. The ^1^H-MRS results showed that there were significant differences in metabolites (mI, *p* = 0.021; Cho, *p* = 0.008) in the left cerebellum between the two groups, as shown in Table 4. There were significant differences in melatonin at both baseline and posttreatment between the two groups (Table 5). Melatonin was increased for patients with ISDHK in the JTW group but decreased in the placebo group. No statistically significant differences in ACTH, GABA or adenosine levels were found between the JTW and placebo groups. After JTW treatment, DHKSS scores in the patients with ISDHK were significantly decreased (Table 6). There was a significant difference in CGI scores between the JTW and placebo groups (2.22 ± 0.856 vs. 3.56 ± 0.601, respectively; *p* < 0.001), which meant that an effective improvement in sleep was observed in the patients with ISDHK after treatment.

**TABLE 3:** Analysis and comparison of PSG in the participants at baseline and posttreatment between the JTW and placebo groups.

	Placebo	JTW	*p* Value
Number			
Baseline	60	58	—
Posttreatment	60	58	—
TST (min)			
Baseline	365.07 ± 102.555	366.42 ± 95.712	0.942
Posttreatment	415.98 ± 87.878	393.92 ± 76.324	0.155
SE (%)			
Baseline	69.48 ± 19.524	68.88 ± 17.827	0.865
Posttreatment	77.95 ± 15.262	73.59 ± 13.580	0.110
ArI (/hr)			
Baseline	10.54 ± 5.072	10.01 ± 5.243	0.580
Posttreatment	10.01 ± 4.861	9.79 ± 4.789	0.808
REM (min)			
Baseline	55.97 ± 31.611	49.48 ± 24.540	0.225
Posttreatment	67.93 ± 29.923	59.25 ± 29.874	0.123
REM (%)			
Baseline	10.70 ± 6.154	9.31 ± 4.530	0.173
Posttreatment	12.71 ± 5.383	10.98 ± 5.386	0.088
N1 (min)			
Baseline	23.60 ± 12.695	25.30 ± 12.935	0.479
Posttreatment	22.80 ± 10.868	23.77 ± 10.859	0.632
N1 (%)			
Baseline	4.44 ± 2.250	4.71 ± 2.277	0.520
Posttreatment	4.28 ± 1.985	4.44 ± 2.015	0.676
N2 (min)			
Baseline	229.23 ± 70.975	238.35 ± 68.123	0.485
Posttreatment	262.06 ± 64.002	251.70 ± 54.957	0.356
N2 (%)			
Baseline	43.60 ± 13.335	44.82 ± 12.868	0.619
Posttreatment	49.18 ± 11.633	47.02 ± 9.932	0.290
N3 (min)			
Baseline	56.28 ± 36.913	53.36 ± 30.740	0.648
Posttreatment	63.20 ± 32.516	59.19 ± 28.202	0.483
N3 (%)			
Baseline	10.73 ± 7.100	10.05 ± 5.919	0.578
Posttreatment	11.81 ± 5.961	12.11 ± 8.603	0.830

TST = total sleep time; SE = sleep efficiency; ArI = microarousal index; REM = rapid eye movement; N1: sleep stage 1; N2: sleep stage 2; N3: sleep stage 3.

**TABLE 4:** Analysis and comparison of ^1^H-MRS in the participants at baseline and posttreatment between the JTW and placebo groups.

	Placebo	JTW	*p* Value
Number			
Baseline	60	58	—
Posttreatment	60	58	—
Left frontal lobe			
GSH			
Baseline	2.98 ± 1.222	2.67 ± 0.485	0.082
Posttreatment	2.96 ± 1.048	2.68 ± 0.504	0.069
mI			
Baseline	8.45 ± 3.349	7.93 ± 1.137	0.264
Posttreatment	8.15 ± 3.052	7.96 ± 1.341	0.672
Cho			
Baseline	2.29 ± 0.798	2.19 ± 0.316	0.351
Posttreatment	2.22 ± 0.821	2.50 ± 2.517	0.421
NAA			
Baseline	14.33 ± 4.723	13.64 ± 1.088	0.281
Posttreatment	13.82 ± 4.355	13.74 ± 1.182	0.888
Cr			
Baseline	10.48 ± 3.556	9.97 ± 0.819	0.285
Posttreatment	9.99 ± 2.934	9.88 ± 0.828	0.777
Glx			
Baseline	19.26 ± 6.753	17.74 ± 1.995	0.103
Posttreatment	17.88 ± 4.931	17.72 ± 2.300	0.828
Right frontal lobe			
GSH			
Baseline	3.01 ± 0.582	3.08 ± 1.230	0.659
Posttreatment	3.15 ± 1.515	2.90 ± 0.586	0.242
mI			
Baseline	8.09 ± 1.557	8.52 ± 2.543	0.272
Posttreatment	8.10 ± 3.009	8.09 ± 1.433	0.984
Cho			
Baseline	2.10 ± 0.298	2.28 ± 0.748	0.071
Posttreatment	2.18 ± 0.834	2.15 ± 0.315	0.816
NAA			
Baseline	14.48 ± 1.014	14.96 ± 4.813	0.449
Posttreatment	14.39 ± 4.186	14.39 ± 1.287	0.989
Cr			
Baseline	10.48 ± 1.098	11.02 ± 3.547	0.262
Posttreatment	10.42 ± 3.608	10.32 ± 0.813	0.837
Glx			
Baseline	20.00 ± 2.189	20.80 ± 6.993	0.398
Posttreatment	19.97 ± 7.090	19.63 ± 2.146	0.727
Left hippocampus			
GSH			
Baseline	3.56 ± 0.866	3.41 ± 0.857	0.393
Posttreatment	3.57 ± 0.889	3.64 ± 0.785	0.662
mI			
Baseline	14.08 ± 2.050	13.89 ± 2.259	0.650
Posttreatment	13.29 ± 2.451	14.01 ± 2.019	0.089
Cho			
Baseline	3.49 ± 0.397	3.43 ± 0.467	0.490
Posttreatment	3.36 ± 0.465	3.50 ± 0.482	0.115
NAA			
Baseline	10.99 ± 1.521	10.69 ± 1.509	0.316
Posttreatment	10.56 ± 1.451	11.02 ± 1.547	0.104
Cr			
Baseline	10.47 ± 1.544	10.18 ± 1.268	0.285
Posttreatment	10.08 ± 1.312	10.28 ± 1.227	0.395
Glx			
Baseline	23.96 ± 3.467	23.29 ± 3.916	0.350
Posttreatment	23.71 ± 3.973	22.65 ± 4.678	0.190
Right hippocampus			
GSH			
Baseline	3.57 ± 0.889	3.38 ± 0.931	0.283
Posttreatment	3.58 ± 1.127	3.93 ± 2.680	0.357
mI			
Baseline	14.01 ± 2.544	13.42 ± 1.774	0.161
Posttreatment	12.93 ± 3.096	14.19 ± 5.732	0.139
Cho			
Baseline	3.37 ± 0.424	3.32 ± 0.459	0.561
Posttreatment	3.42 ± 0.986	3.57 ± 1.461	0.510
NAA			
Baseline	10.36 ± 1.459	10.10 ± 1.460	0.342
Posttreatment	10.81 ± 3.607	10.93 ± 5.523	0.887
Cr			
Baseline	10.11 ± 1.571	9.79 ± 1.659	0.294
Posttreatment	10.23 ± 3.064	10.42 ± 4.601	0.804
Glx			
Baseline	23.71 ± 3.581	22.65 ± 3.090	0.098
Posttreatment	23.37 ± 7.710	23.68 ± 9.435	0.846
Left cerebellum			
GSH			
Baseline	4.38 ± 0.823	4.11 ± 1.184	0.170
Posttreatment	3.95 ± 0.911	4.21 ± 0.919	0.126
mI			
Baseline	13.38 ± 2.065	13.47 ± 1.879	0.822
Posttreatment	12.48 ± 2.449	13.47 ± 2.094	0.021
Cho			
Baseline	3.94 ± 0.448	3.87 ± 0.621	0.483
Posttreatment	3.65 ± 0.562	3.96 ± 0.657	0.008
NAA			
Baseline	15.09 ± 1.232	14.71 ± 1.464	0.141
Posttreatment	14.36 ± 1.740	14.61 ± 2.349	0.525
Cr			
Baseline	15.29 ± 2.483	14.42 ± 2.845	0.086
Posttreatment	13.82 ± 2.987	14.76 ± 3.102	0.098
Glx			
Baseline	24.12 ± 2.075	23.24 ± 3.186	0.088
Posttreatment	22.46 ± 3.355	22.74 ± 4.947	0.722
Right cerebellum			
GSH			
Baseline	4.35 ± 0.749	4.29 ± 1.031	0.732
Posttreatment	4.63 ± 2.172	4.19 ± 0.772	0.146
mI			
Baseline	13.25 ± 1.356	13.64 ± 3.918	0.467
Posttreatment	12.95 ± 2.046	12.99 ± 2.344	0.919
Cho			
Baseline	4.01 ± 0.453	4.12 ± 1.213	0.514
Posttreatment	3.79 ± 0.551	3.98 ± 0.558	0.065
NAA			
Baseline	14.97 ± 1.239	15.27 ± 4.539	0.612
Posttreatment	14.64 ± 1.785	15.00 ± 1.775	0.276
Cr			
Baseline	15.39 ± 2.159	15.26 ± 4.359	0.835
Posttreatment	14.35 ± 2.431	14.90 ± 2.466	0.230
Glx			
Baseline	24.03 ± 2.787	23.83 ± 7.879	0.855
Posttreatment	22.86 ± 4.660	22.57 ± 4.131	0.729
Anterior cingulate gyrus			
GSH			
Baseline	2.96 ± 0.559	3.06 ± 1.158	0.571
Posttreatment	2.98 ± 1.362	2.82 ± 0.448	0.394
mI			
Baseline	8.17 ± 1.075	8.96 ± 3.593	0.105
Posttreatment	8.38 ± 2.939	8.35 ± 1.206	0.928
Cho			
Baseline	2.41 ± 0.357	2.66 ± 1.002	0.075
Posttreatment	2.50 ± 0.873	2.45 ± 0.337	0.713
NAA			
Baseline	12.09 ± 1.095	12.62 ± 4.949	0.422
Posttreatment	12.08 ± 3.788	11.87 ± 0.999	0.680
Cr			
Baseline	9.72 ± 1.085	10.30 ± 3.609	0.236
Posttreatment	9.91 ± 3.345	9.60 ± 0.920	0.501
Glx			
Baseline	21.55 ± 2.262	21.76 ± 8.333	0.851
Posttreatment	21.12 ± 7.058	20.42 ± 3.154	0.495

NAA = N-acetylaspartate; Cho = choline; Cr = creatine, Glx = glutamine and glutamate, mI = myoinositol; GSH, glutathione.

**TABLE 5:** Blood tests in the participants at baseline and posttreatment in the JTW and placebo groups.

	Placebo	JTW	*p* Value
Number			
Baseline	64	64	—
Posttreatment	64	64	—
ACTH (ng/ml)			
Baseline	23.29 ± 13.752	21.39 ± 10.533	0.385
Posttreatment	27.00 ± 16.513	28.98 ± 14.460	0.470
Adenosine (ng/ml)			
Baseline	11.55 ± 20.622	7.84 ± 22.396	0.331
Posttreatment	9.10 ± 14.636	8.94 ± 13.174	0.951
Melatonin (ng/L)			
Baseline	339.60 ± 192.216	210.95 ± 163.753	0.000
Posttreatment	219.59 ± 169.045	339.09 ± 256.894	0.004
GABA (μmol/L)			
Baseline	18.26 ± 11.164	19.32 ± 54.061	0.879
Posttreatment	20.88 ± 23.856	19.98 ± 13.896	0.796

GABA = gamma-aminobutyric acid; ACTH = adrenocorticotropic hormone.

**TABLE 6:** Comparison of DHKSS in the participants at baseline and posttreatment between the JTW and placebo groups.

	Placebo	JTW	*p* Value
Number			
Baseline	64	64	—
Posttreatment	64	64	—
Main symptoms			
Baseline	12.87 ± 0.596	13.73 ± 0.591	0.642
Posttreatment	11.55 ± 0.625	8.39 ± 0.659	0.001
Accompanying symptoms			
Baseline	17.40 ± 1.127	20.06 ± 1.095	0.091
Posttreatment	15.53 ± 1.062	11.10 ± 0.932	0.002
Total score			
Baseline	30.27 ± 1.413	33.78 ± 1.383	0.131
Posttreatment	27.07 ± 1.362	19.49 ± 1.334	0.001

### 3.4 Safety evaluation

All adverse events were documented during the study period. No adverse events occurred in either the JTW or placebo groups. Additionally, no obvious abnormalities were observed in the evaluation of routine blood tests and blood biochemical tests before and after the trial (Table 7).

**TABLE 7:** Blood routine test and blood biochemical test in the participants at baseline and posttreatment between the JTW and placebo groups.

	Placebo	JTW	*p* Value
Number			
Baseline	64	64	—
Posttreatment	64	64	—
Erythrocyte (×10^12^/L)			
Baseline	4.70 ± 0.447	4.69 ± 0.513	0.924
Posttreatment	4.73 ± 0.434	4.71 ± 0.498	0.818
Hb (g/L)			
Baseline	142.55 ± 14.669	143.19 ± 15.085	0.808
Posttreatment	142.97 ± 14.352	143.25 ± 13.862	0.910
Leukocyte (×10^9^/L)			
Baseline	6.21 ± 1.525	6.16 ± 1.381	0.867
Posttreatment	5.95 ± 1.429	6.06 ± 1.334	0.640
Thrombocyte (×10^9^/L)			
Baseline	234.78 ± 58.047	231.99 ± 64.793	0.798
Posttreatment	233.83 ± 66.176	234.66 ± 51.839	0.937
ALT (U/L)			
Baseline	21.03 ± 12.291	20.44 ± 10.924	0.773
Posttreatment	22.81 ± 18.252	20.58 ± 10.772	0.402
AST (U/L)			
Baseline	23.84 ± 8.927	24.05 ± 6.835	0.886
Posttreatment	24.14 ± 9.707	23.89 ± 8.386	0.876
Urea nitrogen (mmol/L)			
Baseline	4.86 ± 1.291	4.66 ± 1.122	0.363
Posttreatment	4.96 ± 1.191	5.17 ± 4.581	0.728
Creatinine (umol/L)			
Baseline	66.53 ± 15.009	65.20 ± 13.364	0.598
Posttreatment	66.84 ± 15.325	65.50 ± 11.484	0.577
Serum potassium (mmol/L)			
Baseline	4.08 ± 0.215	4.13 ± 0.273	0.191
Posttreatment	4.08 ± 0.218	4.09 ± 0.254	0.914
Serum natrium (mmol/L)			
Baseline	139.63 ± 1.759	139.78 ± 1.890	0.629
Posttreatment	140.14 ± 1.572	140.39 ± 1.619	0.377
Chloropenia (mmol/L)			
Baseline	104.42 ± 2.122	104.63 ± 2.082	0.586
Posttreatment	105.17 ± 1.865	105.08 ± 1.970	0.783

AST = glutamic-oxalacetic transaminase; ALT = glutamic-pyruvic transaminase.

## 4 Discussion

To our knowledge, this study was the first to demonstrate that JTW had an effect on symptom relief for patients with ISDHK through a double-blind, randomized, placebo-controlled single-center clinical trial. The primary finding was that JTW was superior to placebo for symptom relief in patients with ISDHK. Unfortunately, no changes were observed in the PSG measures in the treatment group relative to the placebo group. Alterations in the other secondary outcomes, including better CGI scores, decreased DHKSS scores, increased melatonin levels and metabolic changes in the left cerebellum, were observed for patients with ISDHK in the JTW group relative to the placebo group. In terms of safety, JTW had no noticeable adverse effects relative to placebo.

JTW, first appearing in an old classical text of ancient Chinese medicine, has been commonly used for centuries for the management of the ISDHK in Eastern countries. Numerous clinical studies conducted in China have shown that improved PSQI scores were found in patients with ISDHK who were treated with JTW (Yang, 2016; Luo, 2018). A systematic review of the English and Chinese literature demonstrated that oral CHM (including JTW), whether used as monotherapy or adjuvant therapy, could improve subjective sleep quality and quantity for people suffering from insomnia (Ni et al., 2015). Additionally, the review found that the efficacy of CHM in the medium and long term was better than that of Western medicinal drugs and placebo in terms of PSQI scores. Similar to previous studies, effective symptom relief was also observed in ISDHK patients after JTW treatment in this study, and no adverse events occurred in either the JTW or placebo groups. Given that numerous previous studies for CHM were reported to have at least one domain with a high risk of bias, the present study protocol, described in our previous study, was designed in strict accordance with the requirements of RCTs. The results of this study could provide support for the use of JTW as an evidence-based medicine for treating ISDHK. Furthermore, many preclinical studies have also showed that JTW has sedative-hypnotic effects (Quan et al., 2010; Sun et al., 2018; Ni et al., 2019). Unfortunately, no changes were observed in PSG measures after treatment in this clinical study, although PSG is considered the gold standard for evaluating sleep disorders. However, there was an animal experiment showed that JTW could improve sleep time and quality mainly by increasing long-term NREM sleep and reducing the conversion times between NREM sleep and wake (Zeng et al., 2022). It was also demonstrated that JTW reduced the amount of wakefulness, increased the time of NREM sleep and REM sleep in our previous animal experiment (Lin et al., 2022).

The pathophysiology of sleep disorders may involve “hyperarousal” caused by abnormal circadian rhythms (such as melatonin secretion and adenosine receptors), GABA pathways, endocrine factors (high cortisol), and so on (Sarris et al., 2011). In the present study, adenosine, melatonin, GABA and ACTH in blood samples were assessed. The results demonstrated that only melatonin levels were found to be significantly different after JTW treatment, while no changes were found in terms of ACTH, GABA or adenosine. Melatonin is an important endogenous hormone secreted in darkness, is produced by the pineal gland, and can affect sleep homeostasis (Cho et al., 2021). A previous study showed that decreased secretion of melatonin was probably related to the mechanism of insomnia (Takaesu et al., 2015). In addition, melatonin has become commonly known as a supplemental sleep aid. There is meta-analytic evidence that treatment with exogenous melatonin has positive effects on sleep quality (Fatemeh et al., 2021). Exogenous melatonin reduces sleep onset latency and increases total sleep time, whereas it has little if any effect on sleep efficiency (Li et al., 2019). Melatonin levels tended to increase in the JTW group in this study, which may be helpful in elucidating the underlying mechanisms of JTW.

Previous clinical studies have reported that various brain regions showed abnormalities that were related to insomnia; however, they failed to reach an agreement in any converging anatomical or functional region (Sanjari Moghaddam et al., 2021). The prefrontal lobe, hippocampus, cerebellum and anterior cingulate gyrus were selected in the present study for monitoring metabolic changes by ^1^H MRS. The results showed that there were significant differences in metabolic changes in the left cerebellum between the two groups after treatment but not in these other brain regions. The cerebellum has long been considered essential for motor functions and has recently been thought to be involved in various nonmotor functions (Herzfeld et al., 2015; Gao et al., 2018). Some studies have found that the cerebellum is involved in cognition, reward, social behaviour, fear conditioning and so on (Lange et al., 2015; Carta et al., 2019). Additionally, the cerebellum is believed to have a potential regulatory role in the sleep-wakefulness transition (Zhang et al., 2020). Cerebellar dysfunction can cause sleep disturbances, and it is believed that the pathogenesis of insomnia disorder is related to cerebellar abnormalities (Desseilles et al., 2008; Joo et al., 2013). In the present study, the levels of mI and Cho in the left cerebellum remained stable in the JTW group. A recent clinical trial confirmed that mI supplementation could improve global sleep quality, subjective sleep quality, and sleep duration during pregnancy ( Mashayekh-Amiri et al., 2022). Therefore, it is suspected that the effect of JTW on insomnia may be related to metabolic homeostasis in the cerebellum. While the reliability of MRS findings in this study needs further clinical and experimental verification.

This study has several limitations. First, our study may be due to the relatively small number of participants, although the chance for a type II statistical error was small according to our sample size calculation. Second, the study period was short, and there was a lack of medium-to long-term follow-up. The long-term safety and efficacy of JTW are unclear and need further study. Third, this study focused only on the pattern of disharmony between the heart and kidney, which is relatively simple, although it is the dominant type of insomnia. Last, this trial was conducted in a single medical center in China, and an examination of the external validity is still needed.

## 5 Conclusion

JTW is one of the most commonly used CHMs to treat ISDHK in clinical settings using TCM, and this clinical trial provides support with evidence-based medicine that JTW had an effect on symptom relief for patients with ISDHK. In addition, JTW was well tolerated and this study did not point to any safety concerns. Further larger-scale and multi-center clinical trials on JTW as an effective CHM treatment for managing ISDHK are needed.

## Data Availability

The original contributions presented in the study are included in the article/Supplementary Material; further inquiries can be directed to the corresponding author.
